# Vortioxetine in the treatment of adult patients with major depressive disorder: a meta-analysis of randomized double-blind controlled trials

**DOI:** 10.1186/s12888-014-0276-x

**Published:** 2014-09-27

**Authors:** Asres Berhan, Alex Barker

**Affiliations:** Hawassa University College of Medicine and Health Sciences, P. O. Box: 1560, Hawassa, Ethiopia; Veteran Affairs Medical Center, Iron Mountain, Michigan USA

**Keywords:** Vortioxetine, Major depressive disorder, Meta-analysis, Serotonin, Depression symptoms

## Abstract

**Background:**

Vortioxetine is a novel multimodal compound that has recently been approved by the FDA for the treatment of major depressive disorder (MDD). It is a selective serotonin (5-HT) 3A and 5-HT7 receptor antagonist, 5-HT1B receptor partial agonist, 5-HT1A receptor agonist and inhibitor of serotonin transporters. The objective of this meta-analysis was to evaluate the efficacy and safety of vortioxetine in adults with MDD.

**Methods:**

A literature search was conducted in the databases of PubMed, EMBASE, Cochrane library and HINARI. The meta-analysis was conducted by including randomized controlled trials that assessed the efficacy and safety of vortioxetine in adult patients with MDD. Using the random effects model, which assumes individual studies are estimating different treatment effects, the efficacy and safety of vortioxetine was determined by weighted mean differences (WMDs) and odds ratios (ORs). The findings were considered as statistically significant when the 95% CI of WMDs and ORs did not include 0 and 1, respectively. Heterogeneity testing, meta-regression and sensitivity analysis were also performed.

**Results:**

During the initial literature search about 151 publications were identified. Based on the predetermined inclusion criteria, 7 randomized controlled trials were included. The pooled analysis demonstrated a statistically significant reduction in the Montgomery–Åsberg Depression Rating Scale (MADRS) total score from baseline among patients who were on vortioxetine (WMD = −3.92; 95% CI, −5.258 to −2.581). Furthermore, a statistically significant number of patients with MDD who were on vortioxetine have achieved a greater than or equal to 50% reduction in depression symptoms from baseline. However, a significant number of patients who were on vortioxetine therapy reported more adverse events than patients who were on placebo (overall OR = 1.21; 95% CI, 1.06 to 1.38).

**Conclusions:**

Therapy with vortioxetine was significantly associated with reduction in depression symptoms from baseline compared to placebo. Nevertheless, a significant number of patients who were on vortioxetine therapy have reported more adverse events.

## Background

MDD is one of the leading causes of disability worldwide; it is highly recurrent and the symptoms usually persist for longer period. Despite the availability of many antidepressants (tricyclic antidepressants, monoamine oxidase inhibitors, 5-HT reuptake inhibitors and serotonin and norepinephrine reuptake inhibitors) with different mode of actions, an unmet need exists in treating suboptimal efficacy, remission rate and cognition impairments [1]. Therapy with the currently available antidepressant is also associated with side effects, such as sexual dysfunction, suicide risk and weight gain [2,3]. Furthermore, in young adults, long-term antidepressant use suggested to be associated with an increased risk of type 2 diabetes mellitus and bleeding risk [4,5].

Vortioxetine is a novel multimodal compound that has recently been approved by the Food and Drug Administration (FDA) of the United States of America for the treatment of MDD. This drug is a 5-HT 3A, 5-HT7 and 5HT1D receptor antagonist, 5-HT1B receptor partial agonist, 5-HT1A receptor agonist and inhibitor of the serotonin transporter [6,7]. Vortioxetine showed an extended absorption, a medium clearance, a large volume of distribution and a relatively long elimination half-life in healthy young volunteers [8]. Concomitant therapy with drugs involved in the CYP P450 pathways does not seem to have a statistically significant interactions with vortioxetine [9].

Randomized controlled trials that assessed the efficacy of vortioxetine for the treatment of patients with MDD reported contradictory findings. The efficacy of vortioxetine in a study with a duration of 6 weeks was significantly superior to placebo [10]. Similarly, 3 randomized controlled trials with a duration of 8 weeks reported a significant reduction in depression symptoms [11-13]. However, vortioxetine did not differ significantly from placebo in reducing depression symptoms in one study with a duration of 6 weeks and in two studies with durations of 8 weeks [14-16]. The adverse events reported by the patients in the randomized trials were also inconclusive. Thus the primary aim of this meta-analysis is to evaluate the efficacy and safety of vortioxetine at different doses in the treatment of MDD by including randomized controlled trials.

## Methods

### Search strategy

Literature search was conducted by both authors in the databases of PubMed, EMBASE, Cochrane library, HINARI and Google scholar. Major publishers’ websites (Elsevier Science-Science Direct, Nature Publishing Group, Oxford University Press, PsycARTICLES, Science and Wiley-Blackwell) were searched via HINARI. Our search was further strengthened by searching the reference lists of retrieved articles.

The selected key search terms were: MDD, vortioxetine (Lu AA21004), MADRS, Hamilton Rating Scale for Depression (24 items) (HAM-D24) and adverse events. During searching, the term vortioxetine or Lu AA21004 was alternatively combined with other search terms with the help of Boolean logic (AND, OR and NOT).

### Inclusion criteria and study selection

The predetermined study inclusion criteria were: 1) randomized controlled trials that assessed the efficacy and safety of vortioxetine in adult patients with MDD, and studies that recruited patients with MDD presenting with a current major depressive episode of at least 4 weeks duration with no other concurrent psychiatric disorders and MADRS total score of not less than 22 at screening and baseline visits; 2) studies that reported one of the following efficacy or safety measures: change in MADRS and/or in HAM-D24 total score from baseline, proportion of patients who achieved a ≥50% MADRS and/or HAMD24 score reduction from baseline, and number of patients with adverse events.

Study selection was conducted by both authors independently in two stages: first, the abstracts of all the retrieved articles were reviewed and the studies were grouped as either “eligible for full document review” or “ineligible for full document review”. Then, after full document review of all studies that were grouped as “eligible for full document review” were grouped as either “eligible for meta-analysis” or “ineligible for meta-analysis”.

### Data extraction and study quality assessment

The data extraction was also conducted independently by both authors with a similar data extraction template. From the included studies the following information was abstracted: name of the first author, year of publication, duration of therapy, dose, sample sizes, change in MADRS and HAM-D total score from baseline, the number of patients with ≥ 50% decrease in MADRS and HAM-D total score, number of remitters and number of patients with adverse events.

Risk of bias among the included studies was assessed with the Cochrane risk of bias assessment tools. The key domains were: random sequence generation, allocation concealment, blinding of participants and personnel, blinding of outcome assessment, incomplete outcome data, selective reporting and other bias.

### Data synthesis and statistical analysis

In studies where the standard error (SE) and p-values were reported, for ease of use, we transformed to equivalent standard deviation values. Using the random effects model, the efficacy and safety of vortioxetine was determined with WMDs and ORs. For continues variables (the change in MADRS and HAM-D total score from baseline) the WMDs and 95% confidence intervals (CI) were computed using the inverse variance method. While, the ORs were computed using the Mantel-Haenszel (M-H) method. The findings were considered as statistically significant when the 95% CI of WMDs and ORs did not include 0 and 1, respectively. Due to lack of data and inconsistency of reported adverse events across the included studies, the meta-analysis to assess the association of vortioxetine with specific adverse events was restricted to two variables (nausea and hyperhidrosis).

The heterogeneity among the included studies was assessed with I^2^ statistics; when the value of I^2^ was greater than or equal to 50% it was considered as statistically significant. To assess the possible sources of heterogeneity, subgroup analysis based on the durations of therapy and meta-regression using vortioxetine dose as a covariate were performed. To assess the stability of the pooled values to outliers, sensitivity analyses (leave one study out at a time) were performed. All the statistical analyses were performed using OpenMeta-analyst software [17].

## Results

**Figure 1:**
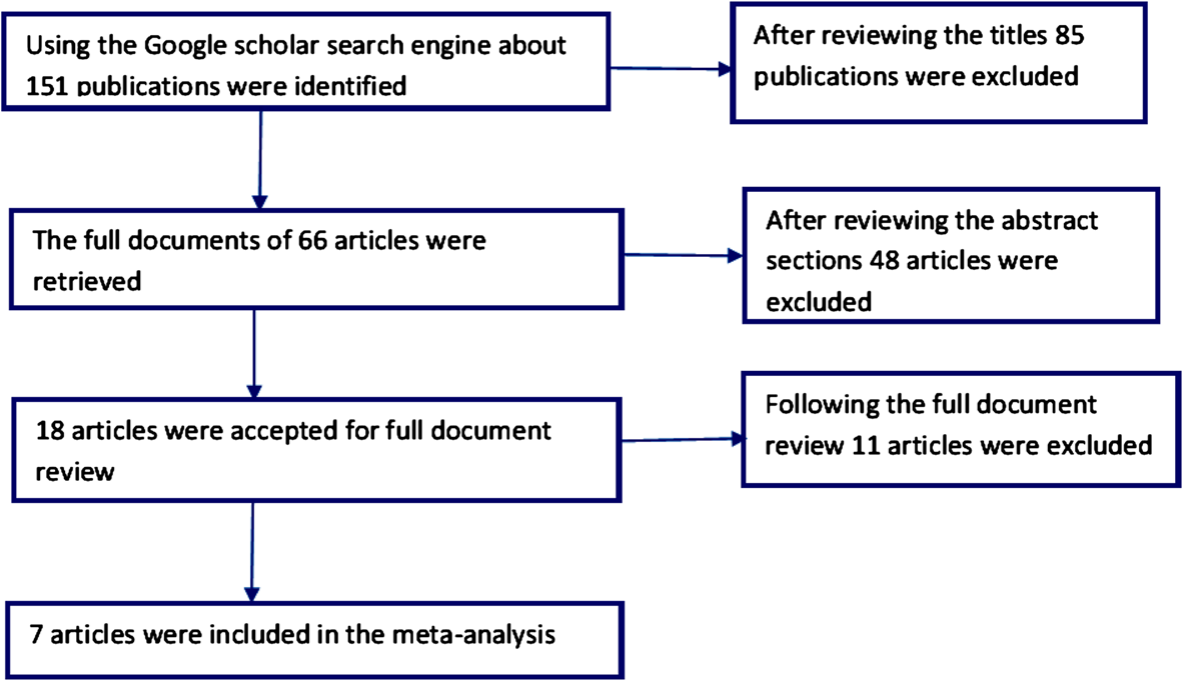
**Flow diagram showing studies selection.**

Using the Google scholar search engine, for the term vortioxetine about 151 publications were identified. Based on the titles proximity to the objective of this study 66 articles were retrieved. In this meta-analysis, in line with the predetermined inclusion criteria, 7 randomized controlled trials were included [10-16] (Figure 1). Except one study [15], all the included studies assessed the efficacy and safety of vortioxetine by randomizing patients in to different study arms with different doses. Reasons for the exclusion of studies were: in vitro studies, animal studies, trial with healthy volunteers and review articles. As presented in Table 1, five of the included studies used duloxetine [11,12,14,16] or venlafaxine [10] as an active reference. While, five of the studies have a duration of therapy of 8 weeks [11-14,16]; the remaining two studies have a duration of therapy of 6 weeks [10,15]. In the selected studies 2,099 patients with MDD were on vortioxetine; 1,130 were on placebo; 705 were on other active drugs (duloxetine or venlafaxine). Though the bias risk assessment demonstrated low risk of bias in randomization and blinding, the presence of other biases (recruitment bias, biases related to the clinical settings etc.) cannot be ruled out.

**Table 1:** **Summary of the included studies in the meta**-**analysis**

**Authors**	**Year**	**Duration**	**Group1/** **Sample**	**Group 2/** **Sample**	**Group 3/** **Sample**	**Group 4/** **Sample**	**Group 5/** **Sample**
Alvarez E et al. [10]	2011	6 weeks	Vortioxetine 5 mg/108	Vortioxetine 10 mg/100	Venlafaxine/113	Placebo/105	---
Baldwin DS et al. [14]	2012	8-week	Vortioxetine 2.5 mg/155	Vortioxetine 5 mg/155	Vortioxetine 10 mg/151	Duloxetine 60 mg/149	Placebo/145
Mahableshwarkar AR et al. [16]	2013	8 weeks	Vortioxetine 2.5 mg/146	Vortioxetine 5 mg/153	Duloxetine 60 mg/149	Placebo/149	---
Katona C et al. [11]	2012	8 weeks	Vortioxetine 5 mg/day/154	Duloxetine/147	Placebo/145	---	---
Jain R et al. [15]	2013	6 wks	Vortioxetine 5 mg/300	Placebo/300	---	---	---
Boulenger JP et al. [12]	2013	8 weeks	Vortioxetine 15 mg/151	Vortioxetine 20 mg/151	Duloxetine/147	Placebo/158	---
Henigsberg N et al. [13]	2012	8 wks	Vortioxetine 1 mg/124	Vortioxetine 5 mg/129	Vortioxetine 10 mg/122	Placebo/128	---

**Figure 2:**
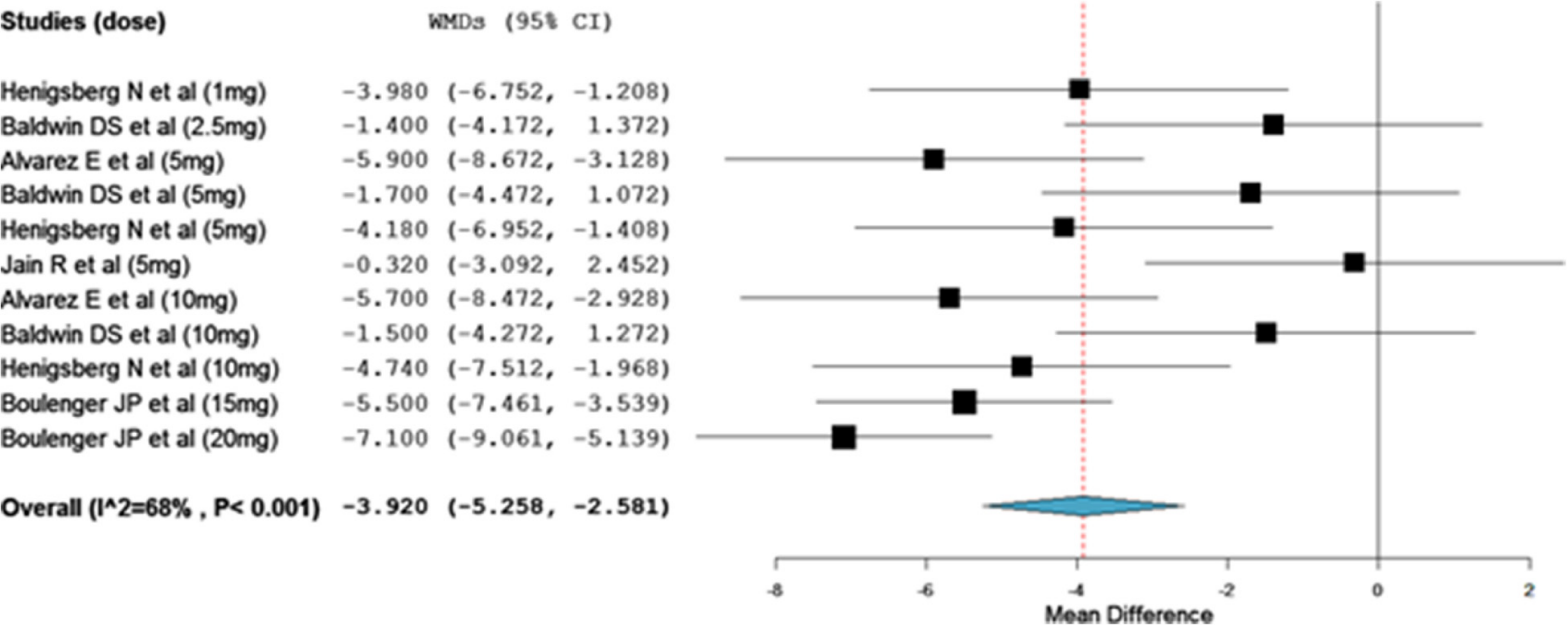
**Weighted mean difference of the change in MADRS total score from baseline.**

**Figure 3:**
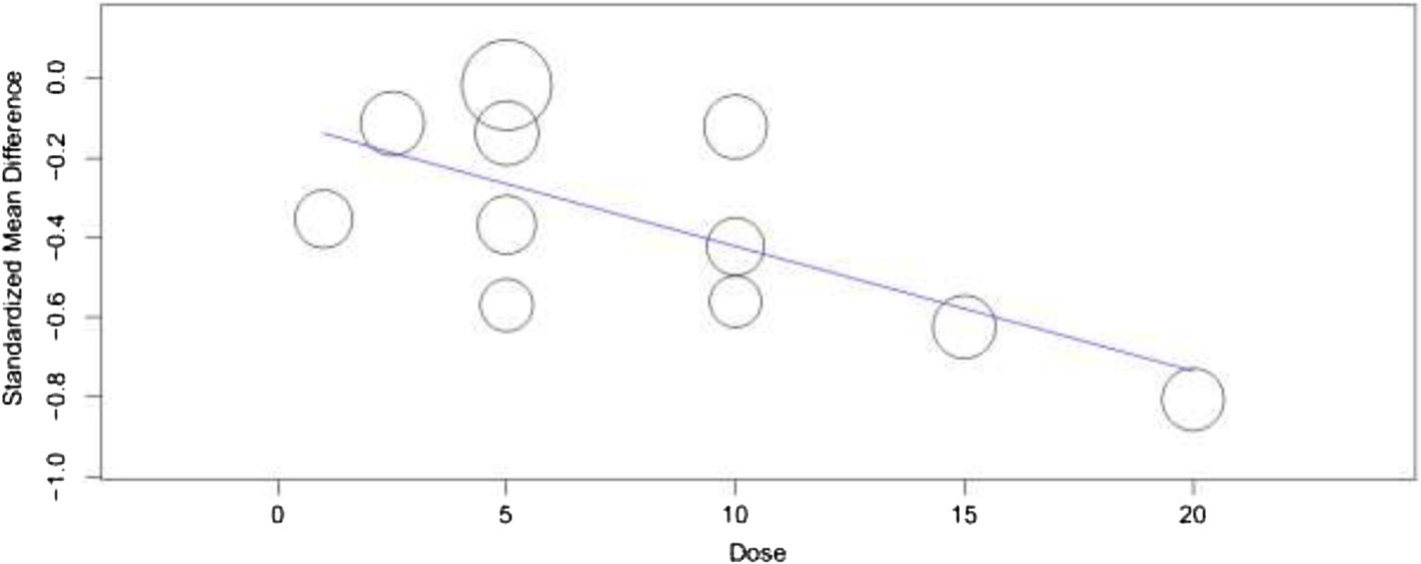
**Change in mean difference in MADRS total score by dose of vortioxetine.**

Figure 2 shows the MADRS mean change from baseline. At the end of interventions, there was a statistically significant reduction in the MADRS total score from baseline among patients who were on vortioxetine as compared with placebo treated patients (WMD = −3.92; 95% CI, −5.258 to −2.581). Though the overall WMD demonstrated the significant reduction in the MADRS total score from baseline in vortioxetine treated groups, in two of the studies the change was not significantly different from placebo treated patients [14,15]. On the other hand, heterogeneity testing revealed the presences of significant heterogeneity among the included studies (I^2^ = 68%). The meta-regression using doses of vortioxetine as covariate showed a statistically significant reduction in MADRS total score with patients who used higher doses of vortioxetine (slope = −0.031; 95% CI, −0053 to −0.009; P = 0.005) (Figure 3). While, the subgroup analysis based on the duration of therapy did not demonstrate a significant difference in therapeutic outcome. Moreover, the sensitivity analysis showed the stability of the overall WMD (WMD do not change significantly when any of the studies is excluded from the analysis).

**Figure 4:**
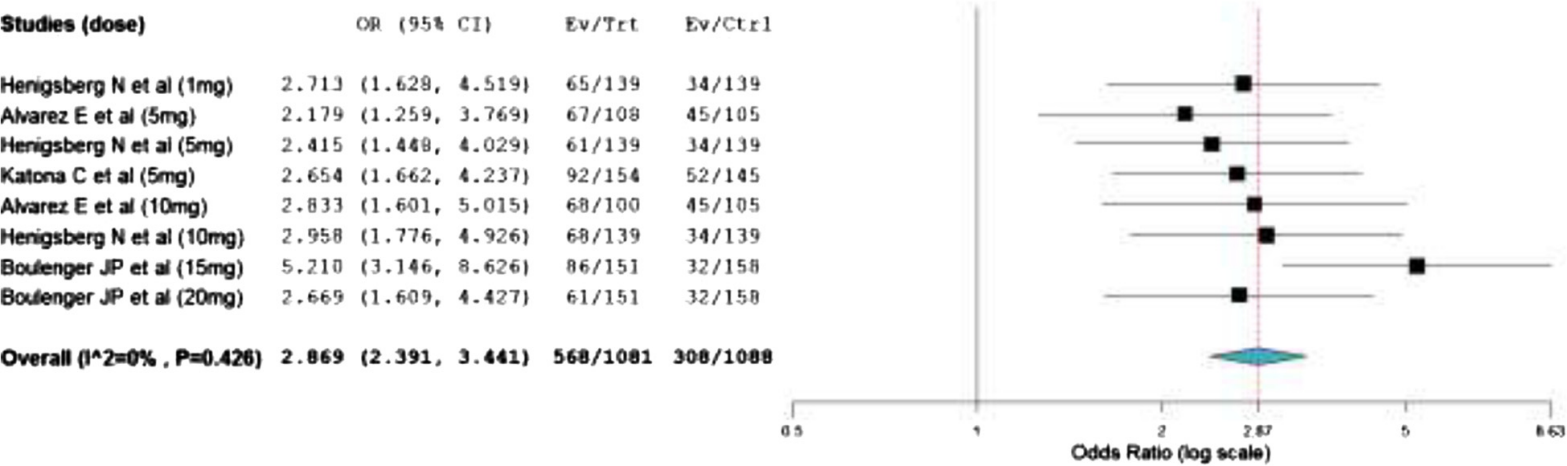
**Mantel-** **Haenszel odds ratio of patients who achieved a** **≥50%** **MADRS reduction from baseline.**

As shown in Figure 4, the odds of vortioxetine treated patients achieving a ≥50% MADRS score reduction from baseline was about 3 times higher than placebo treated patients (Overall OR = 2.87; 95% CI, 2.39 to 3.44). Treatment with vortioxetine was significantly associated with a mean decline in HAM-D24 total score from baseline (WMD = −2.67; 95% CI, −3.96 to −1.38). Consistently, the heterogeneity testing showed the presence of significant inconsistencies among the included studies (I^2^ = 65%). The odds of patients with a decrease in ≥ 50 HAM-D24 total score from baseline was more than 2 fold as compared to placebo treated patients (overall OR = 2.14; 95% CI, 1.54 to 2.97).

**Figure 5:**
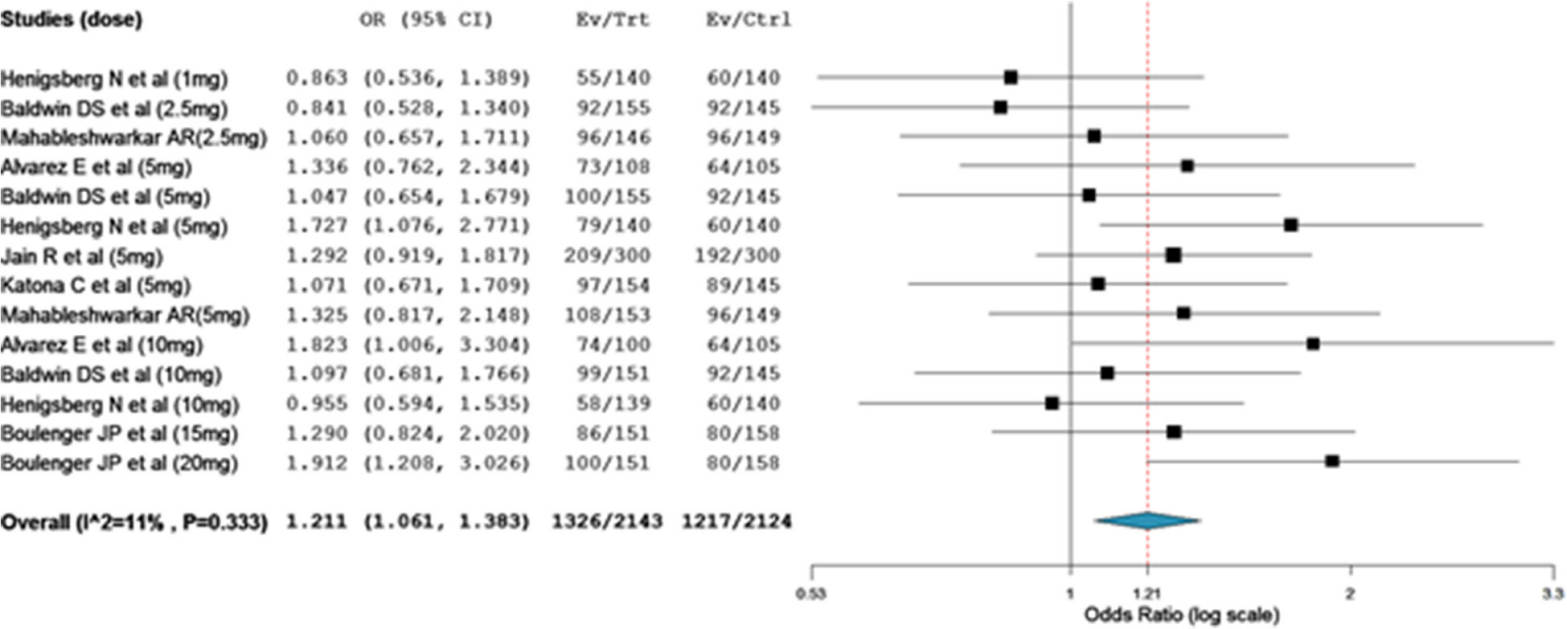
**Mantel-** **Haenszel odds ratio of patients who experienced any adverse events.**

As shown in Figure 5, comparison based on treatment related adverse events demonstrated that a significantly large number of patients treated with vortioxetine experienced more adverse events than patients who were on placebo (overall OR = 1.21; 95% CI, 1.06 to 1.38). The odds of patients who experienced nausea was about 3 fold higher among patients who were on vortioxetine as compared to placebo treated patients (overall OR = 2.89; 95% CI, 2.40 to 3.48). While, the number of patients who were on vortioxetine and experienced hyperhidrosis was no statistically different from placebo treated patients (overall OR = 1.39; 95% CI, 0.72 to 2.66).

## Discussion

This meta-analysis demonstrated the efficacy of vortioxetine in reducing depression symptoms in adult patients with MDD. Based on both depression rating scales (MADRS and HAM-D24) a significant number of patients with MDD who were on vortioxetine therapy have achieved a greater than or equal to 50% depression symptom reduction from baseline. The decrease in depression symptoms seems to be intensified with an increase in the dose of vortioxetine. Furthermore, the efficacy of vortioxetine did not appear to decrease with a long term use; in two multicenter, open-label, flexible-dose extension studies the efficacy of vortioxetine in the treatment of MDD was maintained for about 12 months [18,19]. One of the limitations of most currently available antidepressants is the delay to induce either response or remission (slow onset of action). The lag in onset of antidepressant action is associated with negative consequences, such as increased suicide risk and other deliberate self-harm [20]. However, in vortioxetine treated groups, a significant number of patients with MDD has achieved response or remission within 6 to 8 weeks.

Heterogeneity testing revealed the presence of significant inconsistency among the included studies. While without improvement in the heterogeneity, the sensitivity analysis (leave one study out at a time analysis) showed the stability of the overall WMD. Yet, the reliability of the findings about the efficacy of vortioxetine from this meta-analysis does not seem diminished. This is because; when the number of the included studies in a meta-analysis is small and the heterogeneity is large, the robustness of the finding is best assessed with sensitivity analysis [21].

On the other hand, as compared to placebo treated patients, therapy with vortioxetine was significantly associated with the reported sum total adverse events. Among vortioxetine treated patients with MDD, a statistically significant number of them had experienced nausea. Though primary studies reported the association of vortioxetine with hyperhidrosis, the number of patients with an increased incidences of sweating was not different from placebo treated patients in this meta-analysis. On the other hand, a systematic review that describes the efficacy and safety of vortioxetine concluded no clinically-relevant weight change and suicidal ideation or behavior differences between vortioxetine treated and placebo treated [22]. Moreover, a randomized controlled trial that was designed to assess the effect of single or multiple doses of vortioxetine on cognitive or psychomotor performance concluded no impairment in psychomotor performance [23].

As limitations, first, this meta-analysis noted a significant heterogeneity among the included studies. The most likely explanations for the inconsistency across the included studies could be: the variation in duration of therapy, differences in the baseline disease status, and variation in the doses of vortioxetine used. In support of our last assumption, the meta-regression demonstrated a larger reduction in depression symptoms as the vortioxetine dose increases. Second, during vortioxetine therapy the presence or absence of adverse events that are associated with other currently available antidepressants such as sexual dysfunction, weight change, suicide risk, cognitive impairment are not assessed in this study. Third, studies designed to assess efficacy are usually conducted with relatively smaller sample size and with shorter duration of therapy; thus, meta-analysis by including studies which were not primarily designed to assess adverse events may not have adequate power to assess rare adverse events [24]. Thus, the findings on adverse events related with vortioxetine in this study may not be accurate. Fourth, this study did not assess the possibility of correlation because of multiple dose-placebo comparisons. While, all the included studies were sponsored by pharmaceutical companies. Studies that are sponsored by pharmaceutical companies are likely to be biased by business interests.

## Conclusions

In conclusion vortioxetine was significantly associated with reduction in MADRS total score and HAM-D24 score from baseline. Furthermore, a statistically significant number of patients with MDD who were on vortioxetine have achieved a greater than or equal to 50% reduction in depression symptoms from baseline. Nevertheless, a significant number of patients who were on vortioxetine therapy have experienced adverse events. Thus, its long-term safety and its consistent efficacy in patients with MDD needs further investigations.
